# Brain activation in patients suffering from gambling disorder: an fMRI study using the cue reactivity approach for slot-machine gambling

**DOI:** 10.1192/j.eurpsy.2023.821

**Published:** 2023-07-19

**Authors:** J. Rosenleitner, I. Fuchs-Leitner, R. Kleiser, K. Yazdi

**Affiliations:** Kepler University Hospital, Linz, Austria

## Abstract

**Introduction:**

The relevance of behavioral addictions (like gambling or internet gaming disorder) is growing constantly - not only in clinical practice, but also as a topic in addiction research. Furthermore, behavioral addictions were found to share similar neurobiological mechanisms with substance-use disorders like alcohol or drug addiction. Cue reactivity is a well-established concept to study an important concept in addiction: craving, which denotes the strong desire to consume the addictive substance. For instance, images of alcoholic beverages can induce a strong desire to drink alcohol in patients with alcohol addiction, reflected in brain activation in parts of the reward system and regions specifically involved in craving.

**Objectives:**

In order to extend existing findings to the field of slot machine gambling, we focused on patients suffering from gambling disorder (GD) and who mainly played slot-machines. We investigated neural activation as a response to addiction-related cues (in comparison to neutral cues) using a cue reactivity paradigm.

**Methods:**

To that end, participants with a diagnosis of GD (N = 10) and a group of healthy controls (N = 20) viewed pictures of gambling-related cues (slot machines) as well as neutral cues (ticket vending machines), while brain activation was recorded using functional magnetic resonance imaging (fMRI). Direct comparisons of patients suffering from GD with healthy controls were analyzed for the two different image types (gambling-related vs. neutral) separately.

**Results:**

We found stronger activation in the insular cortex for patients with GD only during presentation of the slot-machine images, but not for the neutral cues. Furthermore, for the slot-machine condition also stronger activation in the dorsal anterior cingulate cortex (dACC) and the supplementary motor area (SMA) was documented for the clinical population but not for the healthy controls.

**Conclusions:**

In line with previous findings, the visual presentation of gambling cues led to stronger brain activations in parts of the reward system (dACC) and in the insula, which plays a crucial role in addictive disorders, especially in craving. Our results further add to the notion that brain areas involved in substance-use disorders might also play an important role in behavioral addictions. Specifically, our findings extend existing results to the research field of slot-machine gambling in the context of craving.

**Disclosure of Interest:**

None Declared

